# Characterization of the Maf family of polymorphic toxins in pathogenic *Neisseria species*

**DOI:** 10.15698/mic2015.03.194

**Published:** 2015-03-02

**Authors:** Anne Jamet, Xavier Nassif

**Affiliations:** 1 Instituto de Microbiologia, Instituto de Medicina Molecular, Faculdade de Medicina, Universidade de Lisboa, Lisbon, Portugal.; 2 Institut Necker Enfants-Malades, 14 rue Maria Helena Vieira Da Silva, CS 61431, 75014 Paris, France.; 3 Université Paris Descartes; Sorbonne Paris Cité, Faculté de Médecine, Paris, France.; 4 INSERM, U1151, Paris, France.; 5 CNRS UMR 8253, Paris, France.

**Keywords:** Neisseria, MafB, toxin, immunity gene

## Abstract

In addition to harmless commensal species, *Neisseria* genus encompasses two pathogenic species, *N. meningitidis* (the meningococcus) and *N. gonorrhoeae* (the gonococcus), which are responsible for meningitis and genital tract infections, respectively. Since the publication of the first *Neisseria* genome in 2000, the presence of several genomic islands (GI) comprising *maf* genes has been intriguing. These GIs account for approximately 2% of the genome of the pathogenic *Neisseria* species and the function of the proteins encoded by *maf* genes remained unknown. We showed that *maf* genes encode a functional toxin-immunity system where MafB is a toxin neutralized by an immunity protein named MafI. A strain can harbor several MafB/MafI modules with distinct toxic activities. MafB toxins are polymorphic toxins with a conserved N-terminal region and a variable C-terminal region. MafB N-terminal regions consist of a signal peptide and a domain named DUF1020 that is only found in the genus *Neisseria*. MafB C-terminal regions are highly polymorphic and encode toxic activities. We evidenced the presence of MafB in the culture supernatant of meningococcal cells and we observed a competitive advantage for a strain overexpressing a MafB toxin. Therefore, we characterized a highly variable family of toxin-immunity modules found in multiple loci in pathogenic *Neisseria* species.

*In silico* analysis, recently revealed that polymorphic toxin-immunity systems are widespread in both Gram-negative and -positive bacteria. In these systems, secreted multi-domain toxins are neutralized by specific cognate anti-toxin named immunity protein. The N-terminal region of the toxin is related to trafficking mode whereas the C-terminal region is hypervariable and carries the toxic activity. Toxin and immunity genes are organized in an operon. Loci also contain cassettes encoding alternative C-termini that could promote diversity of toxic activities through a process of genetic recombination. The polymorphic toxin systems are typically encoded on hypervariable chromosomal islands with characteristics of horizontal gene transfer. These systems are primarily involved in interbacterial competition. For instance, CDI systems, which are a group of polymorphic toxin systems, have been shown to be involved in a contact-dependent inhibition of growth in several Gram-negative bacteria.

*N. meningitidis* is commonly found in the nasopharynx of healthy individuals. In some yet poorly characterized circumstances, the meningococcus can cross the nasopharyngeal mucosal epithelium and cause sepsis or meningitis. *N. gonorrhoeae* is a common cause of sexually transmitted infection. The availability of complete genome sequences for several meningococcal*, *gonococcal and non-pathogenic species strains enabled their comparison to identify relevant genetic variations.

We studied genomic islands containing genes of the multiple adhesin family (*maf*). We identified 3 *maf* genomic islands (MGI-1 to 3) located at the same genomic position in meningococcus and in gonococcus. We also identified 2 additional MGIs (MGI-4 and 5) in gonococcal strains. In contrast, these MGIs were virtually absent in commensal species. A MGI is typified by at least one module of two genes: *mafB* encoding a toxin and *mafI *encoding an immunity protein. MafB proteins exhibit a N-terminal conserved domain of unknown function named DUF1020 (or PF06255 in PFAM Database), which is restricted to *Neisseria* genus, and a C-terminal (CT) hypervariable region. Based on the amino acids alignment of the N-terminal region of MafB proteins, we distinguished 3 classes of MafB. In addition to the *mafB-mafI* module, a *mafA* gene is frequently found immediately upstream of *mafB*. The product of *mafA* gene is predicted to be an outer membrane exposed lipoprotein. It has been shown that gonococcal MafA binds to glycolipids but its precise function remains elusive. Like the DUF1020 domain, *mafA* genes are restricted to the *Neisseria* genus.

To assess the putative function of *mafB-mafI* modules, we expressed four predicted MafB toxins from meningococcal strain 8013 in *E. coli.* Induction of the expression of two MafB proteins was highly toxic for *E. coli* both on Luria-Bertani (LB) agar plates and in liquid LB culture. By cloning only the DNA region encoding the C-terminal domain or the N-terminal domain of MafB toxins, we demonstrated that the toxicity of MafB relies in its C-terminal domain. We showed that the protein encoded by the *mafI* gene located immediately downstream of *mafB* was indeed an immunity protein, able to neutralize its cognate toxin when both proteins are co-expressed. In contrast, there is no cross-protection conferred by a non-cognate immunity protein. We were also able to co-purify a MafB toxin with its cognate immunity protein, which suggests that MafI is likely to inhibit MafB toxicity by a direct interaction. Thus, we have demonstrated that two *mafB-mafI* modules from strain 8013 (*mafBI*_MGI-1NEM8013 _and *mafBI*_MGI-3NEM8013_) are functional toxin-immunity systems when expressed in *E. coli*. Then, we assessed the toxicity of MafB proteins in *Neisseria*. We evidenced that it was impossible to delete the immunity gene of three of the four *mafB* genes of strain 8013 and it was also impossible to insert an ectopic copy of the same three *mafB* genes in the 8013 genome. Thus, three MafB proteins (MafB_MGI-1NEM8013_, MafB1_MGI-2NEM8013_ and MafB_MGI-3NEM8013_) are toxic in their original *Neisseria* strain.

We focused on one MafB toxin from strain 8013 (MafB_MGI-1NEM8013_) that was *in silico* predicted to have a ribonuclease activity cleaving RNA at uridylates (EndoU ribonuclease). We purified this protein and incubated it with a synthetic RNA. A cleavage of this RNA only when it contains uridylates (U) was observed. On the other hand, no cleavage was observed when the cognate purified immunity protein was added to the reaction. Therefore, we characterized the first bacterial EndoU ribonuclease.

Consistent with the fact that MafB sequences contain a signal peptide (SP), we were able to detect MafB in culture supernatants. In addition, we showed that this secretion requires the N-terminal signal peptide. Altogether these data demonstrate that MafB are secreted toxins.

Since other polymorphic toxins have been involved in interbacterial competition, we searched for a role of MafB in competition assays. We used strain 8013 and tested the impact of the overexpression of the four MafB proteins in competition assays. We evidenced a significant competitive advantage for the isolate overexpressing MafB1_MGI-2NEM8013_. This advantage was no longer observed when target cells overexpressed the cognate immunity MafI1_MGI-2NEM8013_. This suggests that MafB1_MGI-2NEM8013_ could be employed by strain 8013 to outcompete strains that do not possess the cognate immunity.

**Figure 1:**
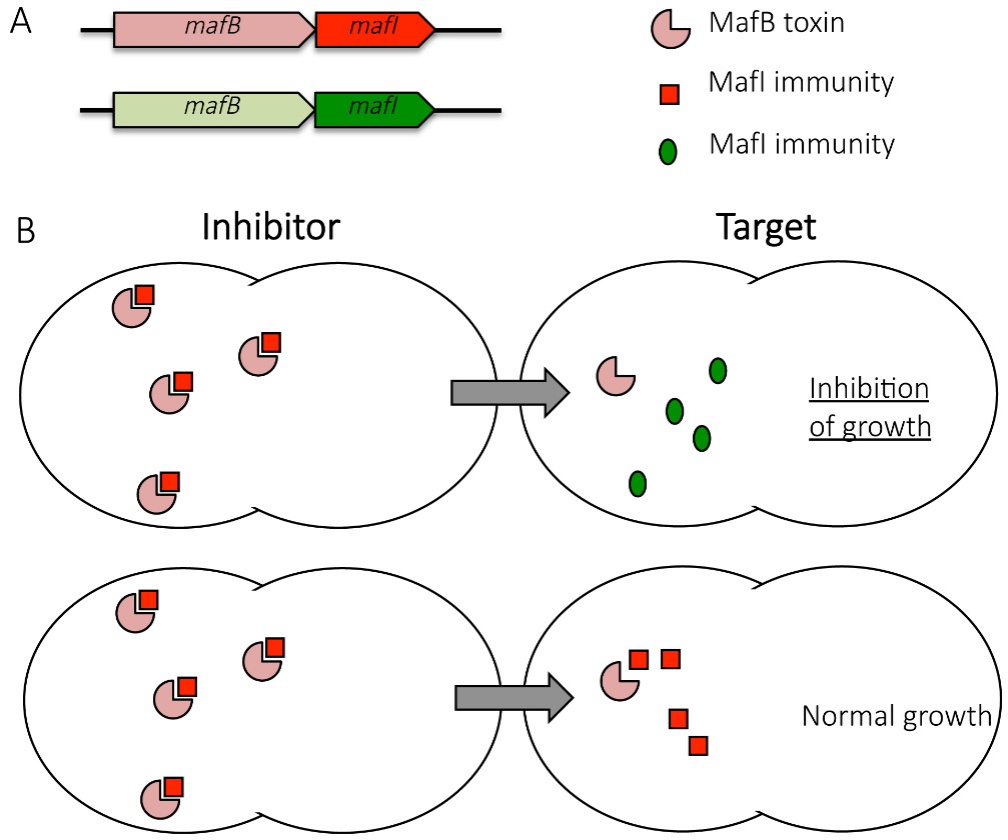
FIGURE 1: MafB toxins are involved in growth inhibition of competitor cells. **(A)** Genomic organization of *mafB* and *mafI* genes. A *maf* locus contains at least one *mafB* gene encoding a toxin and one *mafI* gene encoding the cognate immunity. **(B) **Competition between an inhibitor cell expressing a MafB toxin and a target cell that either lacks or expresses the cognate immunity protein. MafI immunity proteins (blue squares) encoded by *mafI* gene found immediately downstream of *mafB* gene neutralize cognate toxins (red three-quarter circles) whereas other MafI immunity proteins (green oval) do not confer protection.

In addition to the *maf* loci, meningococcal genomes contain other toxin systems (such as bacteriocins and CDI systems). It is therefore difficult to predict the evolution of a complex community when each strain may produce its own repertoire of toxins and immunity proteins.

Exploring the role and the interplay between these toxin-immunity systems *in vivo* should be the aim of future studies.

